# Analgesic, anti-inflammatory and acute oral toxicity profile of leaf and bark extracts of *Albizia procera*

**DOI:** 10.1186/s12906-021-03497-7

**Published:** 2022-02-25

**Authors:** Sangeetha Mani, Chamundeeswari Duraipandian, Saravana Babu Chidambaram

**Affiliations:** 1 Dept of Pharmacognosy, Sri Ramachandra Faculty of Pharmacy, Sri Ramachandra Institute of Higher Education and Research, Porur, Chennai, 600116 India; 2 Dept of Pharmacology, JSS College of Pharmacy, JSS Academy of Higher Education & Research, Mysuru, Karnataka 570015 India

**Keywords:** *Albizia procera*, Acute oral toxicity, Formalin test, Carrageenan, Cotton pellet granuloma, Herbal medicine

## Abstract

**Background:**

Pain and inflammation are associatory events in cancer, diabetes, cardiovascular diseases, arthritis and other chronic diseases. Corticosteroids, non-steroidal anti-inflammatory drugs exert potential side effects on long term use. This study was aimed to investigate the acute oral toxicity, anti-inflammatory and analgesic activities of leaf and bark extracts of *Albizia procera* in experimental animal models.

**Methods:**

Ethyl acetate, ethanol, and hydroalcoholic extracts of *Albizia procera* (leaf and bark) were subjected for acute oral toxicity, anti-inflammatory and analgesic screening. Carrageenan and cotton pellet granuloma models were used to assess acute and chronic anti-inflammatory effects, respectively. Intraplanar formalin test was used to assess the analgesic activity.

**Results:**

All the extracts of *Albizia procera* were found to be well-tolerated up to 2000 mg/kg in female rats. Ethanolic leaf (ETLE) and bark (ETBE) of *Albizia procera* showed anti-inflammatory actions. But, only ETBE produced significant protection in chronic inflammation and analgesic activity.

**Conclusion:**

In summary, *Albizia procera* possess significant anti-inflammatory and analgesic properties. This study adds evidence on the traditional use of *Albizia procera* plant for treating painful inflammatory disorders.

## Background

Herbal and herbo-mineral preparations are popular in the traditional medical systems. The safety and efficacy of medicinal plants in the treatment of chronic inflammatory diseases are indisputable [1]. According to the World Health Organization (WHO) survey, majority of people (> 80%) in developing countries depend on traditional medicines for all primary health issues [2]. The major advantages of herbal medicines are safety and efficacy with no or less adverse effects, even on long term use [3]. In many instances, herbal medicines take a longer time to produce appreciable therapeutic effects. This mandates long term studies to provide scientific evidence on the safety and efficacy. Moreover, the therapeutic doses of herbal medicines are selected based on the toxicity profile of medicinal plants. Hence, the toxicity information of medicinal plants is also mandatory before the efficacy investigation of medicinal plants which were indicated for potential therapeutic benefits in traditional practice [4, 5]. Thus, the primary aim of the toxicological evaluation is to determine the safety, and human usability of plant and also to provide hints on the safer therapeutic dose [6].

Inflammation is an immunological response provoked as a defense mechanism against microbes, infections, cell or tissue injury, toxins etc. Physiological levels of inflammatory reactions, which are self-regulated and self- controlled and are critical for cellular functions and survival but chronic and lethal concentrations lead to severe damage of visceral organs such as brain, heart, lungs, liver, kidneys, pancreas, reproductive systems etc. Uncontrolled inflammatory response or chronic activation of immune cells leads to many life style diseases like rheumatoid arthritis, diabetes, hypertension, obesity, etc. [7, 8]. Chronic inflammation of any part of the body and organs are serious complications to the health system of a human being. Although corticosteroids and non-steroidal anti-inflammatory agents suppress inflammation but impart potent unwanted side effects on long term use [9]. Therefore, there is a need to search safer medicine, particularly from natural origin, for inflammatory disorders. Carrageenan (Kappa) and cotton pellets are commonly used for the induction of inflammation in laboratory rodent models [10]. These phlogistic agents mimic similar pathogenic mechanisms (like the release of histamine, serotonin & kinins; biosynthesis of prostaglandins; activation of protease & lysosomal enzymes; and migration of leukocytes & interleukins) of human inflammatory diseases [10, 11].

*Albizia procera* (Family: Fabaceae), commonly known as White siris and Konda vagai in tamil language, widely grow in India, Nepal, Indonesia, Philippines and Northern Australia. *Albizia procera* is a protein-rich fodder used to feed cattle, camels and elephants in South Asia and Philippines. Leaves of the tree contains α-spinasterol, hentriacontane and hexacosanol. Bark contains pterocarpan - demethylmedicarpin, biochanin A, formononetin, genistein, daidzein and ß-sitosterol. Seeds of *Albizia procera* contains procerogenin-A, mechaerinic acid, proceric acids, proceranin A, oleanolic acid and saponin [12, 13]. A bark decoction is given in chronic inflammatory conditions, including rheumatism [13], but till date there is no scientific evidence of its ethnopharmacological uses. The historical backgrounds of analgesic medicinal plants are opium poppy plants. Most of drugs used to treat inflammation including the natural opium poppy plants reduce vulnerable pain sensation in humans and animals [14, 15]. However, these agents possess addiction behavior, and multiple adverse effects like respiratory depression, drowsiness, reduce gastrointestinal motility, nausea, endocrine disruption, and over-activation of the autonomic system [16]. Hence, there is an urgent need for a safer herbal medicine to treat chronic pain and inflammation.

Many medicinal plant-based medicines possess a promising role in the management of various inflammatory disease [17, 18]. Carrageenan and cotton pellet induced granuloma for inflammation and formalin induced paw pain response are gold standard experiment models to screen the anti-inflammatory and analgesic properties of the new chemical entities. These models cause the release of histamine and serotonin which leads to cause the pain and inflammation in animals [19, 20]. Current research work is focused to evaluate the acute oral toxicity profile, anti-inflammatory and analgesic effects of leaf and bark extracts of *Albizia procera* in rodent models.

## Methods

### Preparation of extracts

The leaves and bark of *Albizia procera* were collected from Tirunelveli district, Tamil Nadu, India. The plant parts were authenticated, and herbarium was deposited in Plant Anatomy Research Centre, Chennai (PARC/2011/2315).

The leaves and bark of *Albizia procera* were freed from earthy matters, sized in to small pieces and separately subjected to successive cold maceration extraction using ethyl acetate, ethanol, and hydro-alcohol (50:50 ratio) *i.e.,* ethyl acetate bark extract (EABE; yield: 1.76%), ethyl acetate leaf extract (EALE; yield: 2.12), ethanolic bark extract (ETBE; yield: 4.5%), ethanolic leaf extract (ETLE; yield: 3.39%), hydro-alcoholic bark extract (HABE; yield: 9.50%), and hydro-alcoholic leaf extract (HALE; yield:6.20%).

### Animal husbandry and ethical approval

Healthy female (125–150 g) and male (160–180 g) Sprague Dawley rats, and Swiss albino mice (either sex; 25–30 g) were procured from M/s. Biogene, Bengaluru (CPCSEA Reg. No. 971/bc/06/ CPCSEA). Animals were maintained in GLP standard facility with room (22 ± 3 °C), humidity: 50–70% with 12 h light / 12 h dark cycle and artificial photoperiod. They were fed with rodent pelleted diet (Nutrilab rodent, Tetragon Chemie Pvt. Ltd., India) and mineral water *ad libitum*. The study protocol was approved (IAECXII/SRU/82/2008, IAEC/XX/SRU/152/2010, and IAEC/XXIV/SRU/186/2012) by the Institutional Animal Ethics Committee, Sri Ramachandra University, Chennai.

### Acute oral toxicity

Acute oral toxicity study was performed as per the Organization for Economic Co-operation and Development (OECD) test guideline-423 (OECD-423; Acute toxic class) method with minor modifications [21]. Healthy young female Sprague Dawley rats (125-150 g) were used in this study. Animals were divided in to groups with three animals in each. Group 1 served as control and were treated with 0.3% carboxymethyl cellulose; Group 2–7 were treated with EABE, EALE, ETBE, ETLE, HABE, and HALE, respectively, at 2000 mg/kg, p.o. in overnight fasted rats. The test extracts were administered at single bolus dose using gastric intubation tube and were observed intensively for the next four hours (30 min, 1, 2 and 4 h) for mortality and toxic clinical signs. Then the animals were monitored for morbidity, mortality, toxicity clinical signs twice a day for next 14 days. At the end of the experimental period, the animals were euthanized using excess ketamine dose (100 mg/kg, i.p) and subjected to gross necropsy for detect lesion, if any.

### Carrageenan-induced acute paw edema test

Carrageenan-induced paw inflammation study was carried out as described by Morris [22] with minor modifications. Female SD rats (160–180 g) were used in the study. Animals were divided in to fourteen groups with five animals in each group. Group-1 served as positive (0.1 ml of 1% carrageenan) and received 0.3% carboxy methyl cellulose (CMC; vehicle) control group. Group 2 was administered reference anti-inflammatory drug *i.e.,* diclofenac (25 mg/kg; *p.o.*). Group 3–14 were consisting of EALE (100 and 200 mg/kg; *p.o.*); ETLE (100 and 200 mg/kg; *p.o.*); HALE (100 and 200 mg/kg; *p.o.*); EABE (100 and 200 mg/kg; *p.o.*); ETBE (100 and 200 mg/kg; *p.o.*); and HABE (100 and 200 mg/kg; *p.o.*) treated groups respectively. One percentage of carrageenan (0.1 ml) was injected in the sub-plantar region of the right hind paw of rat for induction of paw inflammation (edema) after one hour of extracts and diclofenac administration. The carrageenan-induced changes in paw volume were measured at different time intervals *i.e.,* 0.5, 1, 2, 3, and 5 h using plethysmometer (Ugo Basile, Italy) [23]. Percentage inhibition of paw edema was calculated using the formula:

$$\%\mathrm{inhibition}=\frac{\mathrm{Mean}\ \mathrm{paw}\ \mathrm{volume}\ \mathrm{of}\ \mathrm{control}-\mathrm{Mean}\ \mathrm{paw}\ \mathrm{volume}\ \mathrm{of}\ \mathrm{test}\ }{\mathrm{Mean}\ \mathrm{Paw}\ \mathrm{volume}\ \mathrm{of}\ \mathrm{control}}\mathrm{X}\ 100$$

### Cotton pellet granuloma formation test

The cotton pellet induced granuloma test was carried out as described by Goldstein [22, 24] with minor modifications. Male SD rats (160-180 g) were divided in to six groups with six in each. Sterilized cotton pellets (20 ± 1 mg) were implanted (bilaterally) subcutaneously at the dorsal region of all group of rats on day −1. Since, ETLE and ETBE showed potential anti-inflammatory activity in the carrageenan model they were considered for further experiments. Group 1 served as positive control and received 0.3% CMC (Vehicle). Group 2 served as reference control and treated with diclofenac (25 mg/kg; *p.o.*). Group 3–6 were administered with ETLE (100 and 200 mg/kg; *p.o.*); and ETBE (100 and 200 mg/kg; *p.o.*), respectively, for 7 days. On day 8, the animals were anaesthetized using ketamine (35 mg/kg, i.p) and the pellets were taken out. The wet pellets were weighted and then dried overnight at 70 °C. The dried weight of cotton pellets was recorded again. The increase in the dry weight of the cotton pellets is as indicator of granuloma formation. The percentage inhibition of granulation formation by the treatment was calculated by using the formula:

$$\%\mathrm{inhibition}=\frac{\left(\mathrm{X}-\mathrm{Y}\right)\ }{\mathrm{X}}\mathrm{X}\ 100$$

Where ‘X’ is the cotton pellet weight in positive control group and ‘Y’ is cotton pellet weight in extracts treated groups.

### Formalin-induced pain response

Formalin-induced nociceptive pain response was carried out as described earlier [25] with minor modifications [26]. Healthy Swiss albino mice (either sex; 25–30 g) were used in the study. Experimental animals were divided in to six groups with six in each. Group 1 served as positive control and received 0.3% CMC as treatment. Group 2 served as reference control and received aspirin at 50 mg/kg, p.o. Group 3–6 served as test groups. Wherein 3rd and 4th groups received ETLE at 100 and 200 mg/kg, p.o., respectively and 5th and 6th groups received ETBE at 100 and 200 mg/kg; *p.o*., respectively. About 20 μl of formalin (2.5% v/v) was injected in the sub-plantar region of the right hind paw of mice to induce nociceptive response [26]. The nociceptive pain response was assessed by measuring the time spent in paw licking (seconds) between 0–5 min (early phase) and 25–30 min (late phase) after injection of formalin. Additionally, pain rate was scored using a scale pattern *i.e.* i) no pain- normal weight-bearing in injected paw; ii) favoring the injected mice paw and resting lightly on the floor or limping; iii) lifting of the injected paw; and iv) licking the injected paw [25].

### Statistical Analysis

Data were expressed as mean ± SEM; Mean difference between groups were analyzed by one-way ANOVA followed by Tukey’s multiple comparison test using Graph pad prism version 5.0 software. *p* ≤ 0.05 was considered as statistically significant.

## Results

### Acute oral toxicity of Albizia procera

**Fig. 1:**
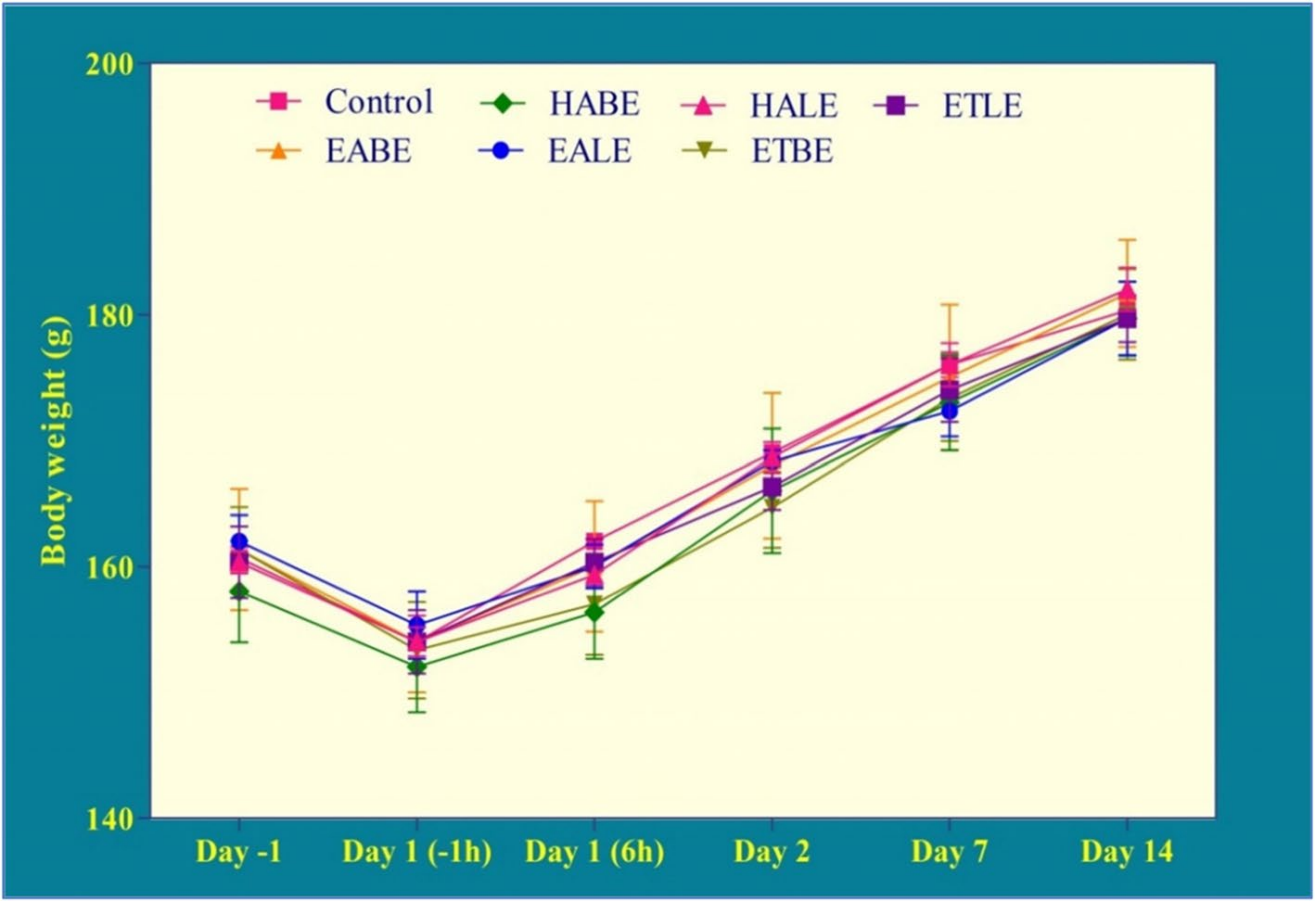
Effects of leaf and bark extracts of *Albizia procera* on animal body weight in acute oral toxicity study. Data were expressed as mean ± SEM. *n* = 3 rats per group

Administration of EABE, EALE, ETBE, ETLE, HABE, and HALE at 2000 mg/kg, p.o. did not cause mortality or morbidity in the overnight fasted female rats. Body weight gain was found to be normal in the test drugs administered groups when compared to the vehicle treated group (Fig. 1**)**. Autonomic profiles *i.e.,* writhing, heart rate, defecation and light reflex; neurological responses *i.e.,* abdominal tone, twitching, grip strength, and limb tone were normal *Albizia procera* extracts (EABE, EALE, ETBE, ETLE, HABE, and HALE) treated groups. In addition, behavioral responses *i.e.,* alertness, irritability, fearfulness, and touch response were also normal. Hence, EABE, EALE, ETBE, ETLE, HABE, and HALE extracts are concluded to be safe at the tested dose. In accordance to Globally Harmonized System, EABE, EALE, ETBE, ETLE, HABE, and HALE are categorized as “category-5” or “unclassified” regimens.

### Ethanolic leaf and bark extract of *Albizia procera* suppressed carrageenan induced inflammation in rats

Carrageenan injection produced persistent increase in paw edema up to 5 h in the vehicle treated group. Administration of ETLE and ETBE reduced paw edema maximum up to 70.89 and 72.79%, respectively. These results were comparable with that of the standard drug diclofenac (Table 1**)**. Ethyl acetate and hydroalcoholic leaf and bark extracts of *Albizia procera* produced no or mild anti-inflammatory effects.

**Table 1:** Effects of leaf and bark extracts of *Albizia procera* on carrageenan-induced paw edema and percentage inhibitions

Group	Treatment	Paw edema [% Inhibition]				
		0.5 h	1 h	2 h	3 h	5 h
I	Positive control	0.48 ± 0.06	0.60 ± 0.07	0.72 ± 0.08	0.82 ± 0.07	0.84 ± 0.08
II	Diclofenac (25 mg/kg)	0.20 ± 0.03** [59.50]	0.19 ± 0.03** [68.39]	0.20 ± 0.02** [71.87]	0.25 ± 0.05** [69.49]	0.29 ± 0.06** [65.27]
III	EALE (100 mg/kg)	0.34 ± 0.04 [29.55]	0.35 ± 0.03* [41.76]	0.37 ± 0.05** [48.75]	0.63 ± 0.08 [23.04]	0.73 ± 0.10 [12.22]
IV	EALE (200 mg/kg)	0.30 ± 0.02 [38.02]	0.40 ± 0.03 [34.28]	0.46 ± 0.04* [36.35]	0.54 ± 0.06 [34.07]	0.55 ± 0.07 [34.61]
V	ETLE (100 mg/kg)	0.27 ± 0.06 [43.80]	0.34 ± 0.03* [44.09]	0.37 ± 0.04** [48.19]	0.50 ± 0.04* [38.73]	0.63 ± 0.11 [24.79]
VI	ETLE (200 mg/kg)	0.20 ± 0.02** [58.68]	0.20 ± 0.02** [66.72]	0.21 ± 0.03** [70.89]	0.33 ± 0.04** [59.07]	0.46 ± 0.03** [44.43]
VII	HALE (100 mg/kg)	0.28 ± 0.02 [41.94]	0.38 ± 0.03 [37.27]	0.44 ± 0.03* [39.14]	0.64 ± 0.05 [21.20]	0.60 ± 0.05 [28.14]
VIII	HALE (200 mg/kg)	0.35 ± 0.05 [28.10]	0.39 ± 0.04 [35.44]	0.45 ± 0.07* [36.77]	0.61 ± 0.08 [25.61]	0.69 ± 0.08 [17.60]
IX	EABE (100 mg/kg)	0.41 ± 0.07 [15.29]	0.50 ± 0.04 [16.97]	0.71 ± 0.01 [0.97]	0.81 ± 0.03 [0.61]	0.83 ± 0.04 [0.12]
X	EABE (200 mg/kg)	0.48 ± 0.06 [0.83]	0.52 ± 0.04 [13.31]	0.67 ± 0.06 [6.13]	0.81 ± 0.05 [0.25]	0.83 ± 0.05 [1.20]
XI	ETBE (100 mg/kg)	0.23 ± 0.02** [52.89]	0.28 ± 0.03** [53.41]	0.31 ± 0.04** [57.38]	0.36 ± 0.04** [56.13]	0.36 ± 0.03** [56.65]
XII	ETBE (200 mg/kg)	0.17 ± 0.03** [65.29]	0.18 ± 0.03** [70.05]	0.21 ± 0.03** [70.47]	0.22 ± 0.02** [72.79]	0.27 ± 0.01** [67.90]
XIII	HABE (100 mg/kg)	0.41 ± 0.04 [16.12]	0.58 ± 0.11 [3.99]	0.71 ± 0.03 [0.97]	0.80 ± 0.08 [2.57]	0.83 ± 0.05 [0.12]
XIV	HABE (200 mg/kg)	0.31 ± 0.03 [36.27]	0.41 ± 0.08 [31.45]	0.65 ± 0.10 [9.19]	0.80 ± 0.08 [1.84]	0.83 ± 0.04 [0.96]

Data were expressed as the standard error of the mean (± SEM). *n* = 6 rats per group. The statistical significance was analyzed by using one way ANOVA followed by Tukey’s multiple comparison tests. Here the symbol ‘*’, and ‘**’ indicates *p*-value <0.05 and *p* < 0.01 respectively when compared to the positive control group

*Abbreviations: EABE* ethyl acetate bark extract, *EALE* ethyl acetate leaf extract, *ETBE* ethanolic bark extract, *ETLE* ethanolic leaf extract, *HABE* hydro-alcoholic bark extract and *HALE* hydro-alcoholic leaf extract

### Effect of leaf and bark extracts of *Albizia procera* in cotton pellet-induced granuloma

**Fig. 2:**
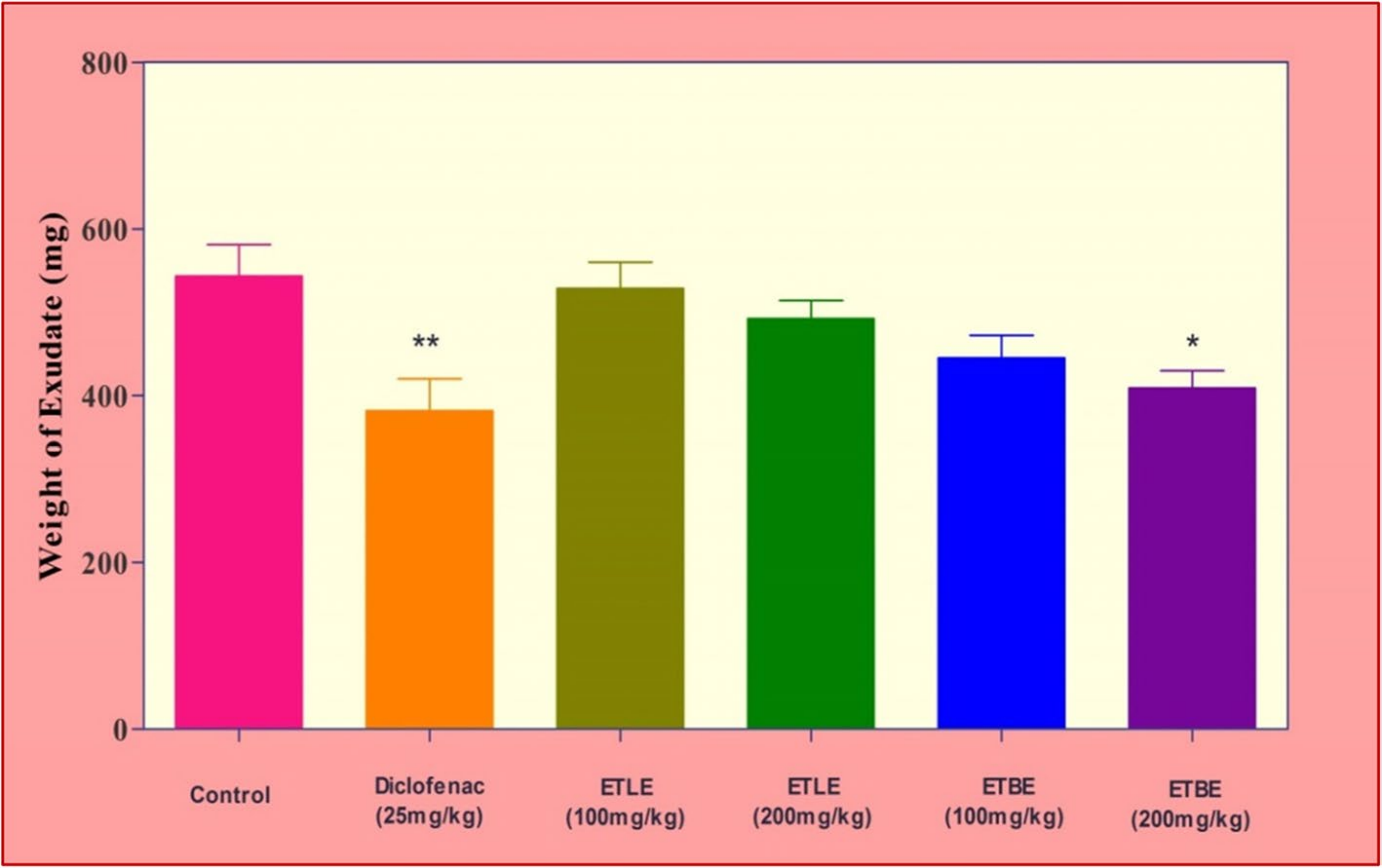
Effects of ETLE and ETBE of *Albizia procera* on exudate weight in cotton pellet granuloma study. Digits in parenthesis indicate dose mg/kg. Data were expressed as the standard error of the mean (± SEM). *n* = 6 rats per group. The statistical significance was analyzed by using one way ANOVA followed by Tukey’s multiple comparison tests. Here the symbol ‘*’, and ‘**’ indicates *p*-value <0.05 and *p* < 0.01, respectively, when compared to the positive control group

Sub-cutaneous implantation of cotton pellet causes granuloma tissue formation in rats. Administration of ETBE produced a dose dependent decrease in decrease in weight of exudate but a significant (*p* < 0.05) decrease was observed at 200 mg/kg, when compared to the vehicle treated group **(**Fig. 2**)**. In addition, ETBE treatment decreased the dry granuloma weights significantly both in 100 and 200 mg/kg, p.o. doses **(**Table 2**)**. The effect of ETBE is comparable with that of the standard drug diclofenac. On the other hand, ETLE did not produce any significant effects on the exudate and granuloma weights.

**Table 2:** Effect of ETLE and ETBE of *Albizia procera* in cotton pellet granuloma formation

Group	Treatment	Weight of Exudate (mg)	Exudate inhibition (%)	Dry weight of granuloma (mg)	Granuloma inhibition (%)
I	Positive control	543.33 ± 37.93	–	140.83 ± 8.44	–
II	Diclofenac (25 mg/kg)	381.50 ± 38.51^**^	42.42	101.50 ± 8.22^*^	38.75
III	ETLE (100 mg/kg)	528.50 ± 31.22	2.81	131.67 ± 9.51	6.96
IV	ETLE (200 mg/kg)	492.33 ± 21.83	10.66	125.33 ± 10.58	12.37
V	ETBE (100 mg/kg)	445.17 ± 27.01	22.05	106.67 ± 4.33^*^	32.03
VI	ETBE (200 mg/kg)	409.00 ± 20.79^*^	32.84	102.17 ± 4.00^*^	37.85

Values were expressed in mean ± SEM; *n* = 6 animals; Significance was analysed using one way ANOVA followed by turkey’s multiple comparison test; *, ** indicates *P* < 0.05 and *P* < 0.01 respectively when compared to positive control

### Effect of leaf and bark extracts of *Albizia procera* in analgesic activity

**Fig. 3:**
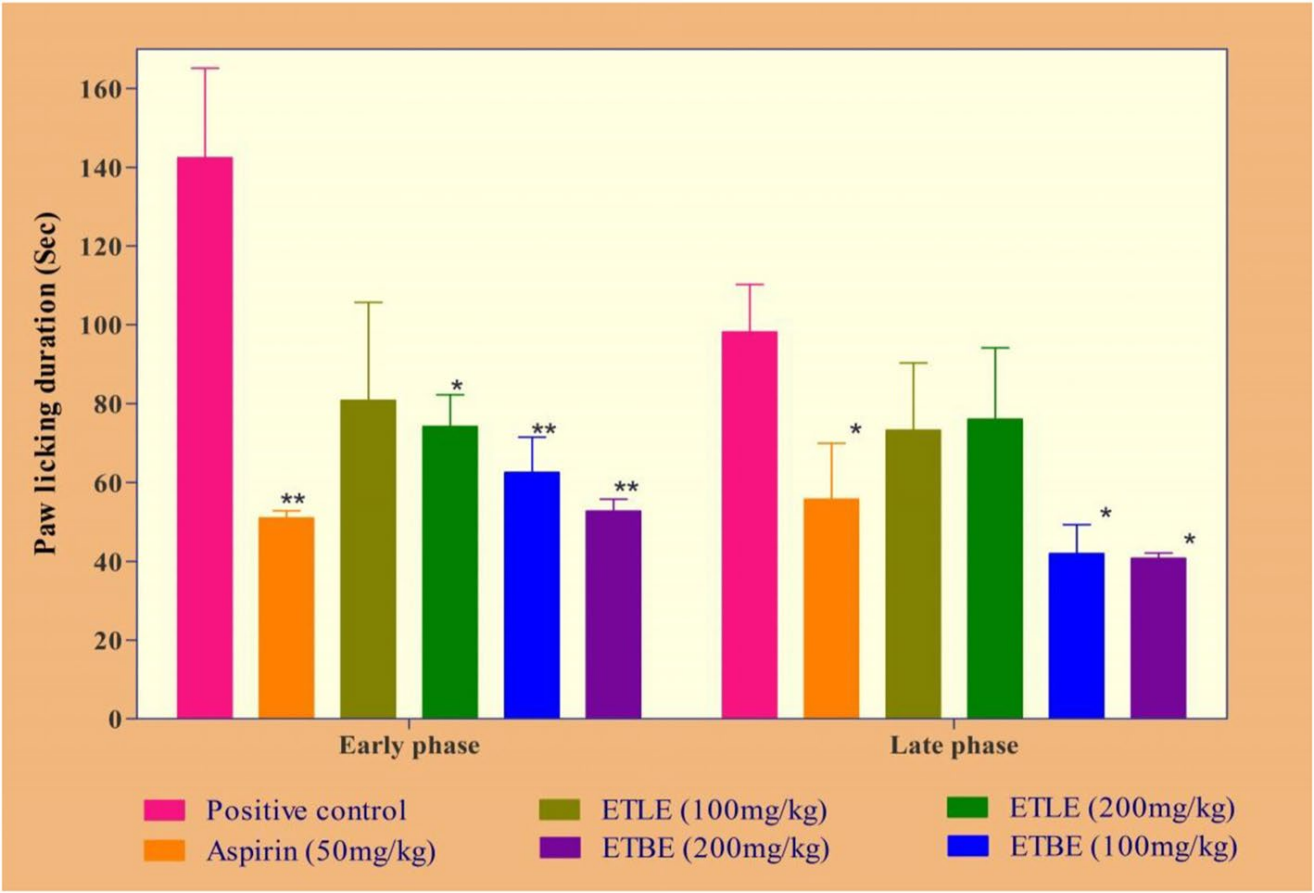
Effects of ETLE and ETBE of *Albizia procera* on formalin-induced paw licking duration. Data were expressed as the standard error of the mean (± SEM). *n* = 6 mice per group. The statistical significance was analyzed by using one way ANOVA followed by Tukey’s multiple comparison tests. Here the symbol ‘*’, and ‘**’ indicates *p*-value <0.05 and *p* < 0.01, respectively, when compared to the positive control group

**Fig. 4:**
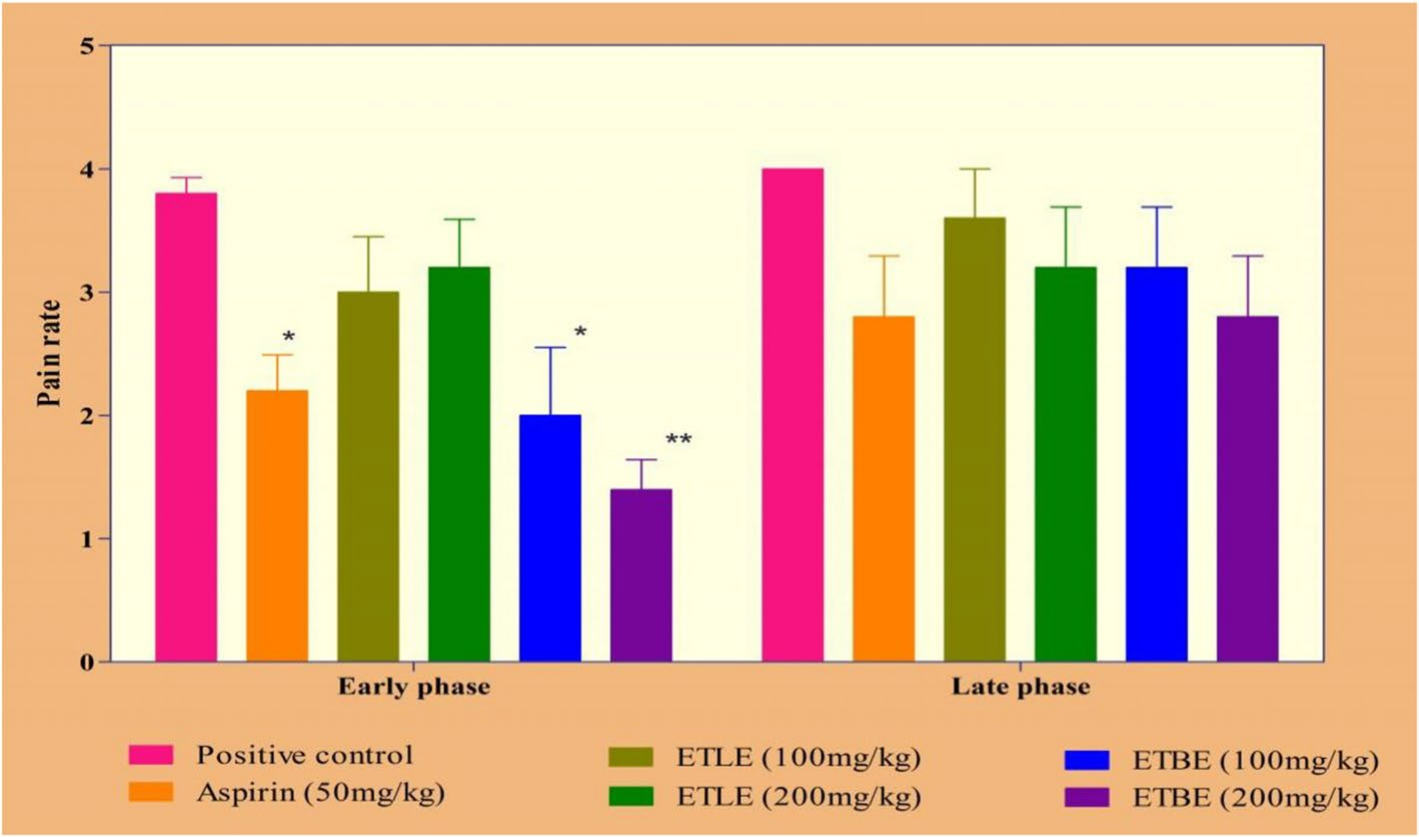
Effects of ETLE and ETBE of *Albizia procera* on formalin-induced pain rate. Data were expressed as the standard error of the mean (± SEM). *n* = 6 mice per group. The statistical significance was analyzed by using one way ANOVA followed by Tukey’s multiple comparison tests. Here the symbol ‘*’, and ‘**’ indicates *p*-value <0.05 and *p* < 0.01, respectively, when compared to the positive control group

ETBE (100 and 200 mg/kg; *p.o.*) treated rats showed a significant (*p* < 0.01) reduction of paw licking duration and pain rate in early phase when compared to positive control group. ETLE at 200 mg/kg also decreased the paw licking duration. Similarly, in the later phase that ETBE significantly (*p* < 0.0o1) decreased the paw licking, but ETLE did not produce any such effect, compared to the positive control group. The effects of ETBE is comparable with diclofenac (Fig. 3**).** On the other hand, ETBE produced a dose dependent decrease in pain rate on the early phase of the experiment, but a non-significant decrease in later phase when compared to the positive control **(**Fig. 4**)**.

## Discussion

EABE, ETBE, HABE, EALE, ETLE, and HALE at 2000 mg/kg is found to be well tolerated in the acute oral toxicity tests in rats. Ethanolic leaf and bark extracts of *Albizia procera* showed potential anti-inflammatory and analgesic activities. ETLE and ETBE of *Albizia procera* showed potential anti-inflammatory action in carrageenan-induced paw edema in rats. The effect of ETBE was found to be better in comparison to ETLE. Additionally, ETLE and ETBE of *Albizia procera* also produced suppression of the amelioration of granuloma tissue formation, but the effects of ETBE was found to be significant. Hence, further studies were planned with ETBE. Intriguingly, ETBE of *Albizia procera* (100 and 200 mg/kg; *p.o.*) showed significant analgesic action against the formalin-induced pain sensation in mice. All these data indicate that *Albizia procera* possess bioactives responsible for anti-inflammatory and analgesic activities.

The treatment of *Albizia procera* has showed an ameliorative effect against the formalin-induced nociceptive pain. Formalin is widely used as an experimental tool for the induction of pain responses in mice. It causes tonic pain sensation based on concentration-dependent [27]. The mechanism of formalin-induced pain is mainly due to the release of histamine, serotonin, and prostaglandins; and up regulation of gene expression of tachykinin receptor 1, 5-hydroxytryptamine receptor 2A, Fos, and opioid receptor-like receptor-1 [28]. Central and peripheral acting analgesic drugs like morphine, aspirin, indomethacin, and diclofenac potentially reduce the pain sensations, but yet these drugs possess significant side effects also [29, 30].

The experimental model of acute inflammation is very commonly developed with carrageenan injection. Carrageenan produces potential inflammation and toxic effects to the muscular and digestive systems. In laboratory animals, it is used for the induction of ulcerative colitis, inflammatory bowel syndrome, rheumatoid arthritis, and colon cancers [31, 32]. The major molecular mechanism of carrageenan induced inflammation is via release of inflammatory and pro-inflammatory mediators like bradykinin, histamine, tachykinins, and free radicals lead to cause tissue injury and inflammation [32, 33]. Treatment with ETLE and ETBE of *Albizia procera* produced a biphasic inhibition of carrageenan-induced paw edema.

In the present study, *Albizia procera* reduced chronic inflammation i.e., granuloma tissue formation in rat model. The formation of granulation is one of the major hallmarks for chronic inflammation with changes in vascularized tissue and it is important components of the wound healing process [34]. Furthermore, infiltrations of neutrophil [35], leukocytes, and T-lymphocytes from the blood stream occurs in granuloma tissue formation [36, 37]. The treatment of ETBE of *Albizia procera* shown to the prevention of granuloma tissue formation against the cotton pellet induced cellular injury.

All these data indicate that *Albizia procera* possess bioactives responsible for anti-inflammatory and analgesic activities and well tolerated biologically.

## Conclusion

The present study provides evidence on the ability of ETBE and ETLE of *Albizia procera* in ameliorating the formalin associated pain sensations and carrageenan-induced paw edema and granuloma tissue formation with relatively safe. Further studies are warranted to determine the active phytoconstituent responsible for its anti-inflammatory, analgesic activity and elucidate the molecular mechanism.

## Data Availability

The datasets used and/or analysed during the current study available from the corresponding author on reasonable request.

## Abbreviations

EABE: Ethyl acetate bark extract
EALE: Ethyl acetate leaf extract
ETBE: Ethanolic bark extract
ETLE: Ethanolic leaf extract
HABE: Hydro-alcoholic bark extract
HALE: Hydro-alcoholic leaf extract
